# 

**Published:** 1878-01

**Authors:** 


CONTENTS.

PAGE.

**CONTRIBUTIONS.**

How to be Sick. BEN. H. PRATT ....309
Paralysis of Liver. P. H. HAYES, M. D. ....312
Only a Boy. B. BROWNE ....314
The Currency. FARMER ....314
Soda. J. B. C. ....315
Surgery vs. Medicine. Prof. WM. TOD HELMUTH, M. D. ....316

**MEDICAL ITEMS AND NEWS.**

Auto-transfusion—Chronic Cystitis—Diarrhœa in Children—Chronic Diarrhœa—Belladonna in Dysentery—Coco as a Remedy for Drowsiness—Phosphorous Pills—Catarrh of Bladder—Tænium Solium in Child 2 years old—The Cock Roach in Dropsy—Quantative Estimation of Urea in Blood—Germs in Disease—Origin of Diphtheria—Dental Caries—Cracked Nipples—Quinia in Small Doses in Malarial Diseases—Sweet Spirits Nitre a Solvent in Salicylic Acid—Tonic for Children—Chafing in Infants—Chafing and Eczema of Vulva—Functional Palpitation—Dropsy—Use of Iron—Chronic Pleurisy with Adhesions—Muriate of Morphia—Opium with those with whom it Disagrees—Leucorrhœa—Chilblains—Triplex Pills—Podophyllin Pills—Solutions of Quinine for Hypodermic Use—Constipation and Hemorrhoids—Dysmenorrhœa—Medical Value of Jaborandi—Experiment on the Development of the Tænia Solium in Man—Chloride of Calcium—Use of Carbonic Acid Gas at Vichy—Bromium of Ammonium in Hay Fever—Treatment of Neuralgia—Smoking Arsenic in Pthisis Pulmonalis ....318 to 326

**OUR CORRESPONDENCE.**

Four Letters with Answers ....326

**HOUSEHOLD HINTS AND RECIPES.**

Liquid Shoe Polish—To Make Shoes Water-tight—To Clean Kerosene Vessels—A Good Varnish—To Remove Silver Stains ....326

**EDITORIAL.**

Diphtheria ....327

**EDITORIAL CHIT-CHAT.**

A New Year—A Pointer—Second Marriages—Fritz—One Handed Women—The Index—Florists—Loaned Newspapers—Cabinet of Brains—Musical Instruments—Poor Jim—Spiral Shirt Studs—Stealing a Camel ....329 to 330

